# Effect of Sound Preference on Loudness Tolerance and Preferred Listening Levels Using Personal Listening Devices

**DOI:** 10.3390/audiolres15030068

**Published:** 2025-06-11

**Authors:** Yula C. Serpanos, Thomas DiBlasi, Jasmin Butler

**Affiliations:** 1 Department of Communication Sciences and Disorders, Adelphi University, Hy Weinberg Center, 158 Cambridge Avenue, Room 122, Garden City, NY 11530, USA; jasminbutler@mail.adelphi.edu; 2 New York Doctor of Audiology (AuD) Consortium, Garden City, NY 11530, USA; 3 Clinical Psychology Doctoral Program LIU Post, Long Island University-Post, Brookville, NY 11548, USA; thomas.diblasi@liu.edu

**Keywords:** loudness tolerance level (LTL), preferred listening level (PLL), personal listening devices (PLDs), hearing health counseling

## Abstract

**Background/Objectives:** This study examined the effect of sound preference on loudness tolerance (LTLs) and preferred listening levels (PLLs) using personal listening devices (PLDs). The implication of this relationship on hearing health promotion counseling and practices using PLDs is discussed. **Methods:** Participants were 50 individuals, aged 21 to 90 years, with normal hearing or hearing loss. Listeners rated several sound samples (i.e., music, running speech, and machinery noise) played through a PLD using earphones according to their sound preference (i.e., enjoyable, acceptable, and unpleasant) and then self-adjusted the volume setting to their LTL and PLL for a sound sample in each sound preference category. **Results:** Most listeners judged music (70%) as *enjoyable*, running speech (54%) as *acceptable*, and machinery noise (84%) as *unpleasant.* No significant differences were found in LTLs according to sound preference, but PLLs for enjoyable sounds occurred at significantly higher levels compared with those deemed acceptable or unpleasant. **Conclusions:** Listeners using PLDs perceived LTLs and PLLs differently according to their sound preferences. PLLs occurred at significantly higher volumes for sounds deemed enjoyable when using PLDs. The implication is that hearing health counseling should include information to PLD users on the potential of altered loudness perception with enjoyable sounds, which may lead to higher and riskier PLD listening levels.

## 1. Introduction

Exposure to excessive sound levels is one of the most common and preventable causes of acquired hearing loss [1]. Notably, the wide availability and common recreational use of mobile personal listening devices (PLDs, e.g., smartphones, MP3/4 players) is linked to hearing loss from repeated and prolonged listening at high volume levels through earphones [2]. Global estimates suggest that half of those aged 12 to 35 years listen with PLDs at levels considered unsafe for hearing [3], creating risk for hearing loss in approximately 1.35 billion people [4]. Untreated hearing loss has many potential consequences on an individual’s quality of life and can impact listening and communication, cognition, education, employment, mental health, and social relationships [3,5]. In response to a worldwide concern, the WHO and the International Telecommunication Union (ITU) published the WHO-ITU H.870 Global Standard On Safe Listening Devices And Systems with the aims of regulating sound level exposures using PLDs and reducing their risk for hearing loss [2].

Although many types of audio and audiovisual content are available for listening using PLDs (e.g., music, podcasts, movies, gaming), the previous literature has primarily reported effects on hearing from listening to loud music using mobile devices (e.g., [6,7,8,9]). The popularity of music listening with PLDs led to the term *music-induced hearing loss (MIHL)* to label the damaging effect on the auditory system from exposure to high levels of music (e.g., [9,10,11,12]). There is evidence that adolescents and young adults continue to engage in risk-taking listening behaviors using PLDs despite knowledge of the effects of excessive sound levels on hearing [13].

Several behavioral theories have been proposed as to why people listen to music at high volumes; however, they do not offer a comprehensive explanation as to why people who listen to loud music engage in such behavior. Florentine and colleagues [14] compared loud music listening to addiction. Welch and Fremaux [15] took a biopsychosocial approach to explaining this behavior. In their Conditioning, Adaptation, and Acculturation to Loud Music (CAALM) model, they argued that evolutionary, biological, psychological, social, and cultural factors of listening to loud music maintain the behavior. They described that there may be a biological desire for loud music, given brainstem reflexes [16,17] and increased dopamine levels [18], as well as personality characteristics (e.g., sensation seekers [19]) that may make it more likely to enjoy loud music. Over time, adaptation occurs, the physiological response decreases, and tolerance develops, similar to that of addiction. This means that music needs to be even louder to have the same effect on the body (e.g., arousal). As volume levels increase, it leads to acculturation to loud music—making loud music the norm in society. This is evidenced by business models that promote loud music [20,21] and the frequent locations of loud music: fitness centers [22], sporting events, bars [23], and nightclubs [24]. Given the consistent exposure to loud music throughout society, classical conditioning occurs [25]. Individuals associate loud music with these events and the accompanying benefits, including social cohesion and increase in positive emotions, making them more likely to hold positive attitudes regarding loud music. Importantly, this is a self-reinforcing cycle that leads to continual exposure to loud music.

Some studies have shown that loudness perception is altered for enjoyable sounds, such as with music. For example, music that is well-liked is perceived as less loud by listeners than music that is not liked [26]. Further, the more music is liked, the louder it is preferred [27]. For others, loud music exposure may be an unwanted occurrence causing discomfort and therefore classified as noise [28]. Franklin and colleagues [29,30] determined that measures of loudness tolerance and acceptance of background noise using running speech (referred to as the *acceptable noise level* [ANL]) were unrelated in listeners with normal hearing or hearing loss. The authors concluded that loudness tolerance and the ANL reflect different auditory processes and listening strategies and supported the clinical value of these measures in amplification fittings. Gordon-Hickey et al. [31] compared ANLs for running speech to ANLs for several music samples rated by preference in a group of listeners with normal hearing. ANLs for music were found to be better (smaller) than ANLs for speech and unrelated to music preference. This finding implied a greater acceptance of music as background noise and may support that music is processed differently as background noise than speech [31].

The effect of categorical sound preference on loudness judgments when using PLDs is not known—that is, whether sounds deemed acceptable or unpleasant are tolerated and adjusted differently for volume when using PLDs than those considered enjoyable. The purpose of this study was to determine whether listeners using PLDs perceived loudness tolerance levels (LTLs) and preferred listening levels (PLLs) differently according to their categorical preference of sound (i.e., enjoyable, acceptable, and unpleasant). It was hypothesized that LTLs and PLLs increase as a function of sound preference (i.e., enjoyability) when using PLDs. A further understanding of this relationship may inform hearing health promotion counseling and practices using PLDs.

## 2. Materials and Methods

### 2.1. Participants

This study involving human participants was reviewed and approved by the Institutional Review Board of Adelphi University, Garden City, New York. Using G*Power (Version 3.1), a power analysis indicated that 42 participants were needed while holding for a moderate effect size, alpha of 0.05, power of 0.80, three groups, two measurements, and a correlation of 0.50. Fifty adult individuals were recruited through the university and completed consent forms for participation in this investigation. The participants were 28 females and 22 males, ages 21 to 90 years (mean age = 38.2 years).

### 2.2. Procedures

Hearing assessment was conducted in a sound-treated test booth following standardized protocols to determine the hearing status of participants. An air conduction pure-tone hearing screening was performed in each ear at 25 dB HL from 500 to 8000 Hz [32]. Those who did not pass the screening at 25 dB HL underwent air conduction pure-tone hearing threshold assessment from 500 to 8000 Hz [33]. Participants were seated in a quiet room and asked to listen to several sound samples played through a smartphone device (Apple iPhone 12, Apple Inc., Cupertino, CA, USA; iOS system version: 15.4) using wired earbud earphones (Apple EarPod, Apple Inc.). Listeners placed the earphones themselves, and a visual insertion check was performed by the investigator. No listeners reported discomfort or a loose fit with the earbuds. The sound samples were downloaded by the investigator from the internet through the smartphone and consisted of one random selection each from a category of music (i.e., mainstream pop songs), running speech (i.e., informational and entertainment podcasts), and machinery noise (e.g., construction and drilling sounds). The investigator manipulated switching between each sound sample selection. The listeners were requested to rate the sounds in one of three preferential categories as *enjoyable*, *acceptable*, or *unpleasant*. The sound samples were presented separately for 1 min.

Listeners were then instructed to self-adjust the volume setting of the PLD using the volume toggle attached to the earbud earphones according to their LTL for 1 min of listening to the sound sample that matched their sound preference for each separate category (*enjoyable*, *acceptable*, and *unpleasant*). Re-instruction was provided as necessary. The LTL was defined in the instructions as the highest volume level that could be tolerated without discomfort for 1 min of listening. The same procedure was used to identify the listener’s PLL, defined in the instructions as the volume level they would prefer for 1 min of listening. The volume output reading was gathered by the iPhone internal headphone audio level feature and is reported in decibel (dB) units as was displayed by the device. The maximum sound volume for the device was 102 dB. The LTL and PLL for each sound preference category (i.e., *enjoyable*, *acceptable*, and *unpleasant*) were documented for each participant onto a data spreadsheet. A repeated measures analysis of variance (ANOVA) was used to determine significant differences (*p* < 0.05) by condition and sound preference category (LTLs and PLLs), as well as the interaction between the two. An independent *t*-test was conducted to examine if there was a difference between participants with normal hearing and hearing loss.

## 3. Results

**Figure 1:**
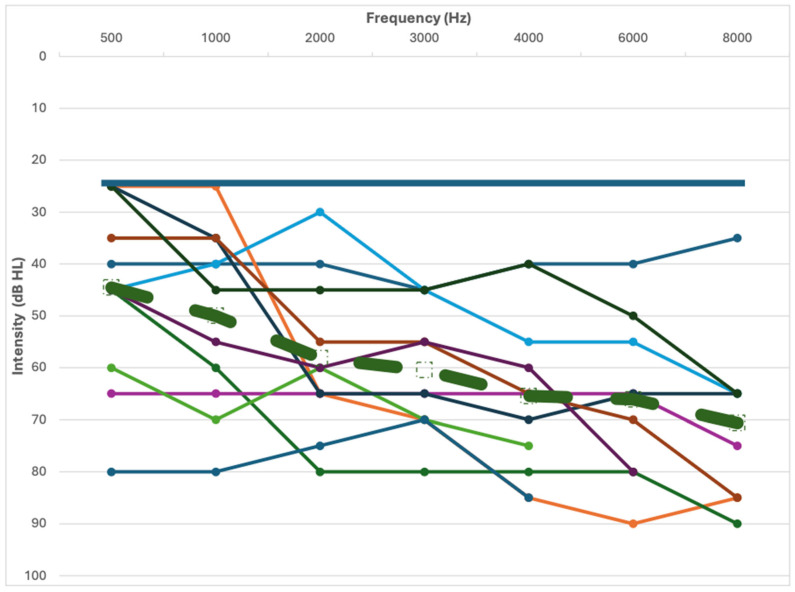
Air conduction pure-tone hearing thresholds are displayed from the poorer ear for 11 participants with hearing loss. Absent thresholds occurred at some high frequencies for three participants due to hearing exceeding equipment limits. Mean thresholds for hearing loss are represented by the thick dashed line. The solid thick line represents the 25 dB HL screening level for normal hearing (*n* = 39).

Hearing outcomes revealed that 39 participants presented with normal hearing < 25 dB HL and 11 with hearing loss > 25 dB HL. The demographics and hearing information for the participants are presented in Table 1 and Figure 1.

Most listeners judged music (70%) as *enjoyable*, running speech (54%) as *acceptable*, and machinery noise (84%) as *unpleasant* (Table 2).

**Figure 2:**
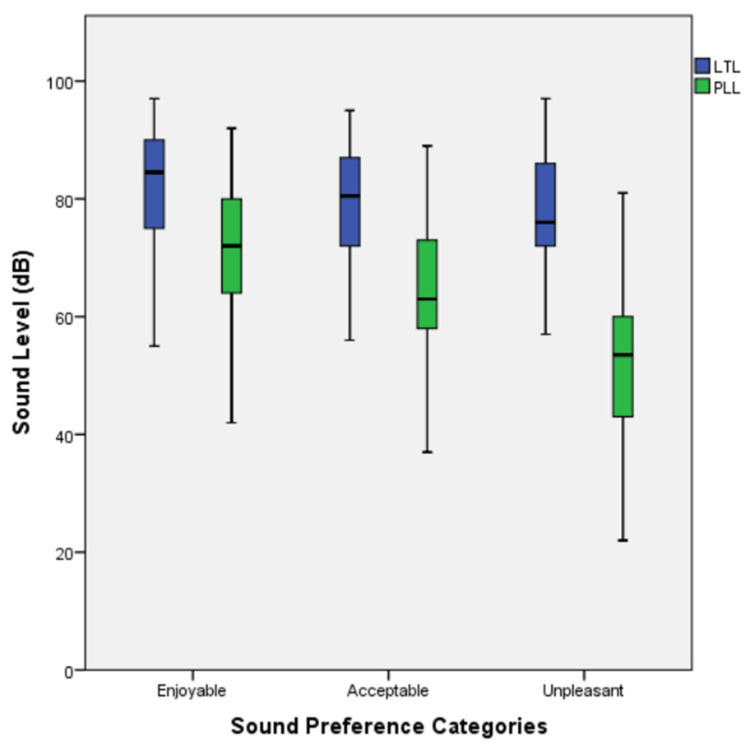
Loudness tolerance levels (LTLs) and preferred listening levels (PLLs) by sound preference category. Sound levels were reported in dB according to the smartphone device internal headphone audio level feature display.

Mean values across the sound preference categories (*enjoyable*, *acceptable*, and *unpleasant*) were 81.4 dB (*SD* = 10.4), 78.1 dB (*SD* = 11), and 76.7 dB (*SD* = 11) for LTLs, and 71.2 dB (*SD* = 11.4), 64.4 dB (*SD* = 12.4), and 50.7 dB (*SD* = 13.2) for PLLs, respectively (Figure 2).

### 3.1. Normal Hearing Compared to Hearing Loss

A comparison of the sound level judgments revealed no significant differences in the sound preference categories for listeners with normal hearing compared to those with hearing loss: *enjoyable* (LTL: *t*(48) = −0.22, *p* = 0.82; PLL: *t*(48) = −1.78, *p* = 0.08); *acceptable* (LTL: *t*(48) = −0.009, *p* = 0.99; PLL: *t*(48) = −1.37, *p* = 0.18); and *unpleasant t*(48) = −0.66, *p* = 0.51; PLL: *t*(48) = 1.18, *p* = 0.24).

### 3.2. Repeated Measures ANOVA

A mixed model for repeated measures ANOVA was conducted to examine the main effect of condition on sound preference category, as well as the interaction effect between condition and sound preference category. There were only two levels of the within-subject factor (sound preference category (i.e., PLL and LTL)), thus Mauchly’s Test of Sphericity could not be performed, as it is only appropriate for three or more levels. Consistent with the power analysis, the Pearson correlation was r(150) = 0.53.

### 3.3. Main Effect of Condition

There was a significant difference between conditions, *F*(2, 147) = 19.50, *p* < 0.001, *η*2 = 0.21 (large effect size). Using the Bonferroni correction, pairwise comparisons indicated a significant difference between the enjoyable condition compared to the acceptable condition (*p* = 0.04), enjoyable compared to the unpleasant condition (*p* < 0.001), and acceptable compared to the unpleasant condition (*p* < 0.001), see Table 3.

### 3.4. Main Effect of Sound Preference Category

There was a significant main effect on sound preference category, *F*(1, 147) = 328.01, *p* < 0.001, *η*2 = 0.69 (large effect size). Post hoc analyses were conducted using the Bonferroni correction. LTLs (*M* = 78.72, *SD* = 10.91) were significantly higher than PLLs (*M* = 62.09, *SD* = 14.97).

### 3.5. Interaction Between Condition and Sound Preference Category

Using Wilks’ Lambda, there was a significant interaction between condition and sound preference category (*F*(1, 147) = 328.01, *p* < 0.001, *η*2 = 0.69 (large effect size)), as such, post hoc analyses were conducted.

#### 3.5.1. Interaction Between Condition and LTL

There was no significant difference between conditions on LTLs. Participants did not rate their sounds judged as enjoyable (*M* = 81.36, *SD* = 10.36) any different than acceptable (*M* = 78.10, *SD* = 11.02; *p* = 0.41) or unpleasant (*M* = 76.68, *SD* = 11.02; *p* = 0.10). Additionally, participants did not rate their LTLs higher for sounds judged as acceptable compared to unpleasant, *p* = 1.00. The Bonferroni correction was applied.

#### 3.5.2. Interaction Between Condition and PLL

Participants rated their PLLs higher for sounds judged as enjoyable (*M* = 71.20, *SD* = 11.39) compared to sounds judged as acceptable (*M* = 64.38, *SD* = 12.39; *p* = 0.02) and unpleasant (*M* = 50.68, *SD* = 13.23; *p* < 0.001). Additionally, participants rated their PLLs higher for sounds judged as acceptable compared to unpleasant, *p* = 0.001. The Bonferroni correction was applied.

#### 3.5.3. LTLs Compared to PLLs

There was a significant interaction effect between condition and sound preference categories *F*(2, 147) = 35.75, *p* < 0.001, *η*2 = 0.33 (large effect size). The Bonferroni correction was applied. LTLs (*M* = 81.36, *SD* = 10.36) were significantly higher than PLLs (*M* = 71.20, *SD* = 11.39) when comparing the enjoyable sound preference, *p* < 0.001. LTLs (*M* = 78.10, *SD* = 11.02) were also significantly higher than PLLs (*M* = 64.38, *SD* = 12.39) when comparing the acceptable sound preference, *p* < 0.001. Lastly, the results were similar for the unpleasant sound preference. LTLs (M = 76.68, SD = 11.02) were significantly higher than PLLs (*M* = 50.68, *SD* = 13.23), *p* < 0.001.

## 4. Discussion

This study evaluated the loudness perception attributes of tolerance and preferred listening behaviors according to sound preference categories (i.e., enjoyable, acceptable, and unpleasant) when using PLDs. These outcomes supported that listeners with or without hearing loss judged their tolerance and preference of loudness listening differently according to their sound preference when using PLDs.

### 4.1. LTLs

Loudness tolerance was not affected by sound preference; there was no significant difference in LTLs among sounds judged categorically as enjoyable, acceptable, or unpleasant in the group of listeners with normal hearing or hearing loss. Interestingly, LTLs were significantly greater than PLLs across all three sound preference conditions. This may suggest that loudness tolerance is a different perceptual process than loudness preference. To our knowledge there are no other studies that directly compared LTL to PLL according to sound preference when using PLDs. However, Franklin et al. [29,30] found no correlation between loudness tolerance and acceptance of background noise and concluded that these measures reflect different aspects of loudness perception. There may be other possible explanations for the differences between LTLs and PLLs and the lack of effect of sound preference category on LTLs. The literature has reported on inconsistencies in measures of loudness tolerance influenced by test instructions and methods [34]. An alternative explanation is that participants view the task of tolerance as a game or challenge. Research supports the notion that individuals frame tasks as games, and when they do so, it leads to increased motivation to perform better on the task [35]. Gamification leads to increased motivation, dopamine [36], and improved results in a variety of contexts [37], including work [38], education [39], health [40], and marketing [41,42]. The thought of “winning the game”, even if they are not directly playing a game, may lead to increased dopamine, and increased motivation to achieve to the highest LTL. This does not mean they are enjoying the volume at that level, and in fact, it could be beyond their level of enjoyment (as evidenced by participants’ PLLs in this study). Instead, regardless of sound preference category, participants are engaging in risk-taking listening behaviors, similar to what is found in gaming [43,44] in order to “win the game” and have a high LTL. Future studies may benefit from examining this further using an experimental design. Additionally, the use of artificial intelligence and large language models may be beneficial in examining this closer [45].

### 4.2. PLLs

Individuals in this study adjusted their preference for listening at significantly higher levels with sounds deemed enjoyable over those considered acceptable or unpleasant. It should be noted that enjoyable sounds were not only identified as music; for example, 15% of listeners categorized running speech formats (such as podcasts) as enjoyable. We are not aware of other studies that evaluated preferred listening when using PLDs according to sound enjoyability, but investigations using music have identified higher PLLs according to music style [10,46].

The mean PLL for this group of participants across sound categories is considered within the safe listening level recommended by the WHO-ITU H.870 Global Standard on Safe Listening Devices and Systems [2]. However, the finding that individuals prefer to use a higher volume setting on their PLDs when listening to enjoyable sounds may cause them to listen at higher levels than what is considered safe. One reason for that is that positive mood can alter perception and make risky behaviors appear less risky [47,48]. When individuals are in a positive mood and recognize the risks involved in a situation, they are more likely to engage in risk aversion [49].

### 4.3. Implications for Hearing Health Counseling

Hearing health intervention strategies using PLDs have been mainly targeted at music listening [9]. Although these are preliminary results, this study demonstrated that sounds perceived as *enjoyable*, music or other types, yielded higher user preferred volume listening levels. It may be important for hearing and other health professionals to counsel the public on safe listening practices with PLDs, emphasizing the term *enjoyable* to reference the perception of sounds (not just music) that can affect how loud people listen and potentially create risk to hearing health. This could be a key counseling approach for hearing health professionals, as individuals using PLDs may not have an accurate loudness judgment for enjoyable sounds, which may cause them to listen at higher levels than what is considered safe; however, if they are made aware of the risk, they might be less likely to listen to enjoyable sounds at higher levels. Common protective strategies recommended for PLD users include using isolator-style earphones and adhering to specific guidelines for safe volume listening levels [9]. For example, the WHO-ITU H.870 Global Standard on Safe Listening Devices and Systems recommends levels below 80 dB for a maximum 40 h duration in a week. But Portnuff [9] suggested that providing maximum volume targets for safe listening using PLDs may be too restrictive, leading some populations such as adolescents to disregard the message.

Importantly, it may not simply be about giving individuals information that listening at high volumes can lead to hearing loss but about making them aware of the risk in the moment (such as by use of mindfulness). While hearing health professionals could recommend that their patients practice mindfulness, as it has shown been to increase awareness and intentional decision making [50], it may also be helpful for hearing health professionals to implement motivational interviewing (MI) to increase individuals’ motivation to change [51]. MI helps individuals resolve ambivalence towards change and has been found to be effective in over 2000 controlled trials [51], including a variety of different settings and issues (e.g., addiction, mental health, physical health, and dental health). In fact, a systematic review found MI was an effective intervention for hearing health professionals to increase hearing aid use in patients [52]. In MI, the practitioner comes from a place of compassion, listening and attempting to elicit change talk from the individual. For example, instead of saying “Why do you listen to enjoyable sounds so loud”, (which would elicit patient sustain talk [i.e., language to maintain their current behavior]) a hearing health professional may ask “What would it take for you to listen to enjoyable sounds lower?” Another example is “What do you think are the negative consequences to listening to your PLD at high volumes?” By eliciting change talk (i.e., language in favor of changing), the individual is demonstrating that they are aware of the information, and are actually “talking themselves into change”, which is more likely to lead to them listening at safe volumes. This is especially important, as Ismail and colleagues [52] described hearing health professionals’ behaviors as inconsistent with MI, tending to be task-oriented and more one-sided conversations, with the hearing health professional predominately speaking. Given this, if hearing health professionals implemented MI, the authors speculate that patients may be more likely to adhere to hearing health professionals’ recommendations.

### 4.4. Strengths and Limitations

This study provided new information on loudness tolerance and preferred listening characteristics by sound preference when using PLDs. Participants with normal hearing and hearing loss were both included, since that is representative of the general population, thus increasing generalizability and external validity. This decision was further supported by the lack of statistical differences between the two groups. However, the statistical tests comparing the groups with normal hearing (*n* = 39) and hearing loss (*n* = 11) are underpowered, limiting meaningful subgroup conclusions.

Several study limitations are identified and should be considered in the context of these findings and for future investigations in this area. There may have been variability in the loudness measures imposed by the device output calibration and the individual self-adjusted sound levels. This study did not calibrate the device output levels but reported user dB levels according to the iPhone internal headphone audio level feature. The limitations of smartphone dB metrics may warrant calibrated output measures in prospective investigations. The procedure of measuring LTLs before PLLs may potentially have created an order effect or perceptual adaptation, potentially elevating PLL values. A standardized protocol was not used to assess the sound preference categories; future studies may include validated tools to improve the generalizability and psychometric strength of these measures. Although there was no statistical difference between normal hearing and hearing loss participants, this may be considered an exploratory finding due to the sample size imbalance, and it could be that differences exist between groups that were not observed in this study. Future studies may benefit from replicating this study with solely normal hearing or hearing loss participants.

## Figures and Tables

**Table 1:** Participant characteristics.

Characteristics	Hearing Loss	Normal Hearing
Age (years)		
Mean	65.5	30.6
*SD*	22.5	13.4
Range	24 to 90	21 to 79
Gender (*n*)		
Females	7	21
Males	4	18
Total	11	39
Pure Tone Average (dB HL)		
Mean	50.5	*
*SD*	14.6	*
Range	35 to 78.3	*

*Note*. Air conduction pure-tone averages were calculated from thresholds (in dB HL) at 500, 1000, and 2000 Hz and are reported for the poorer ear for the participants with hearing loss; *SD* = standard deviation; * pure-tone screening at 25 dB HL was used to determine normal hearing.

**Table 2:** Mean sound preference category judgments by sound sample.

Sound Sample	Preference Category		
	Enjoyable	Acceptable	Unpleasant
Music			
*n*	35	14	1
%	70	28	2
Running Speech			
*n*	15	27	7
%	30	54	14
Machinery Noise			
*n*	0	9	42
%	0	18	84

**Table 3:** Descriptive statistics for sound preference category by condition.

Sound Preference Category by Condition	*M*	*SD*
Enjoyable		
LTL	81.36	10.36
PLL	71.20	11.39
Total	76.28	11.98
Acceptable		
LTL	78.10	11.02
PLL	64.38	12.39
Total	71.25	13.55
Unpleasant		
LTL	76.68	11.02
PLL	50.68	13.23
Total	63.68	17.82

*Note*. Sound preference category broken down by condition.

## Data Availability

Data supporting the results are reported within this article in the figures and tables; further inquiries can be directed at the corresponding author.

## Abbreviations

The following abbreviations are used in this manuscript:

PLD	Personal listening device
LTL	Loudness tolerance level
PLL	Preferred listening level
ANL	acceptable noise level
dB	Decibel
HL	Hearing level
Hz	Hertz
WHO	World Health Organization
SD	Standard deviation
